# Sex-Specific Computational Models of Kidney Function in Patients With Diabetes

**DOI:** 10.3389/fphys.2022.741121

**Published:** 2022-01-26

**Authors:** Sangita Swapnasrita, Aurélie Carlier, Anita T. Layton

**Affiliations:** ^1^Department of Cell Biology-Inspired Tissue Engineering, MERLN Institute for Technology-Inspired Regenerative Medicine, Maastricht University, Maastricht, Netherlands; ^2^Department of Applied Mathematics, University of Waterloo, Waterloo, ON, Canada; ^3^Department of Biology, Cheriton School of Computer Science, School of Pharmacology, University of Waterloo, Waterloo, ON, Canada

**Keywords:** sex differences, diabetes mellitus, SGLT2 inhibitors, diuresis, natriuresis, kaliuresis, sodium transport, glucose transport

## Abstract

The kidney plays an essential role in homeostasis, accomplished through the regulation of pH, electrolytes and fluids, by the building blocks of the kidney, the nephrons. One of the important markers of the proper functioning of a kidney is the glomerular filtration rate. Diabetes is characterized by an enlargement of the glomerular and tubular size of the kidney, affecting the afferent and efferent arteriole resistance and hemodynamics, ultimately leading to chronic kidney disease. We postulate that the diabetes-induced changes in kidney may exhibit significant sex differences as the distribution of renal transporters along the nephron may be markedly different between women and men, as recently shown in rodents. The goals of this study are to (i) analyze how kidney function is altered in male and female patients with diabetes, and (ii) assess the renal effects, in women and men, of an anti-hyperglycemic therapy that inhibits the sodium-glucose cotransporter 2 (SGLT2) in the proximal convoluted tubules. To accomplish these goals, we have developed computational models of kidney function, separate for male and female patients with diabetes. The simulation results indicate that diabetes enhances Na^+^ transport, especially along the proximal tubules and thick ascending limbs, to similar extents in male and female patients, which can be explained by the diabetes-induced increase in glomerular filtration rate. Additionally, we conducted simulations to study the effects of diabetes and SGLT2 inhibition on solute and water transport along the nephrons. Model simulations also suggest that SGLT2 inhibition raises luminal [Cl^–^] at the macula densa, twice as much in males as in females, and could indicate activation of the tubuloglomerular feedback signal. By inducing osmotic diuresis in the proximal tubules, SGLT2 inhibition reduces paracellular transport, eventually leading to diuresis and natriuresis. Those effects on urinary excretion are blunted in women, in part due to their higher distal transport capacity.

## Introduction

In recent years, the role of sex and gender has emerged as a priority area in biological and medical research (Layton, 2021). In particular, there has been increasing evidence that sex has a significant impact on the pathogenesis of metabolic disorders, such as type 2 diabetes (T2D). Diabetes prevalence is currently estimated to be 9.3% (463 million people) worldwide and expected to reach 10.2% (578 million) by 2030 and 10.9% (700 million) by 2045 (Saeedi et al., 2019). In developed countries, T2D is associated with chronic kidney disease (Foley and Collins, 2007) and increases the risk of cardiovascular disease (Koye et al., 2018). Interestingly, sex-specific differences have been reported in the disease prevalence and incidence of diabetes and diabetic kidney disease. Overall, men are predisposed at a higher rate to T2D and a similar prevalence of type 1 diabetes (T1D) compared with premenopausal women. Postmenopausal women, however, have an increased risk of developing glomerular hyperfiltration, diabetic kidney disease, and end-stage kidney disease, compared to age-matched men (Bjornstad and Cherney, 2018; Lovshin et al., 2018).

To understand the origin of the differences in the prevalence in diabetes-induced renal complications between men and women, one must first understand kidney function and its sex differences. The kidney’s nephrons are the centers for filtration of electrolytes and water in the blood and the maintenance of pH. Each human kidney contains about a million nephrons, which are linked to clusters of glomerular capillaries that receive blood from individual afferent arterioles branching off into intra-renal arteries. A portion of that blood passes through the glomeruli and enters the nephron. Along the different segments of the nephrons (namely, the proximal tubules, the loops of Henle, the distal convoluted tubules, the connecting tubules, the collecting ducts), the content of the tubular filtrate undergoes major changes, *via* epithelial transporter-mediated reabsorption or secretion of solutes, and reabsorption of water, to become urine. In both women and men, urine output matches fluid and solute intake as well as waste production. However, major sex differences may be observed in the epithelial transport processes of the kidney.

Sex hormones regulate nearly every tissue and organ, including the kidney (Reckelhoff, 2001; Sabolić et al., 2007; Hilliard et al., 2011; Hatano et al., 2012; Sullivan and Gillis, 2017). Veiras et al. (2017) reported sexually dimorphic patterns in the distribution of renal transporters (electrolyte, channels, and claudins) across the different nephron segments in male and female rodents. Their findings demonstrated that female rat nephrons exhibit lower reabsorption of Na^+^, bicarbonates, and water along the proximal tubules, resulting in a downstream shift of the Na^+^ and water reabsorption in female kidneys. Along the distal nephron segments, the female kidney exhibits a higher abundance of key Na^+^ transporters, relative to male, resulting in similar urine excretion between the sexes. In addition, experimental evidence has shown that uremic toxin transporters also display sex differences (Urakami et al., 1999; Groves et al., 2006; Euteneuer et al., 2019), for example the organic anionic transporter (OAT3) is expressed less in male than female mice, and the opposite is valid for OAT1 (Breljak et al., 2013). Our modeling studies in the male and female rat kidney have previously identified the functional implications of these molecular differences in renal handling of water, electrolytes, and glucose (Li et al., 2018; Hu et al., 2019, 2020) and in renal nitric oxide bioavailability and medullary oxygenation (Chen et al., 2010, 2017; Fry et al., 2014, 2015, 2016). For example, due to their lower Na^+^ activity in the proximal tubule, female rats are able to more rapidly excrete a saline load (Hu et al., 2019).

Some of the sex differences in rodent kidney function may translate to humans. Despite obvious inter-species differences, women and female rats face similar challenges of circulating volume adaptation during pregnancy and lactation. In a recent study, we developed sex-specific models of epithelial transport along the nephrons in the kidney of a man and a woman (Hu et al., 2021). Model simulations indicate that sexual dimorphism in renal transporter patterns similar to that reported in rodents may better prepare women for the heightened demands on the kidney during pregnancy and lactation (Hu et al., 2021). An important open question remains: How do these findings translate to kidney function in diabetes? Indeed, differences have been reported in the prevalence and severity of diabetic kidney disease between women and men (Maric-Bilkan, 2020). Even though the pathogenesis of diabetic kidney disease remains incompletely understood, pathophysiological changes that diabetes induces in the kidneys have been characterized. At the very onset of diabetes, the kidney enlarges and glomerular filtration rate (GFR) becomes supranormal (Mogensen, 1971). Moreover, diabetes induces an alteration in transporter expression: the activity of SGLT2, GLUT2, NKCC, and Na/K-ATPase are upregulated whereas the SGLT1 activity is downregulated, and this in a nephron segment specific manner (Layton et al., 2016a). These structural and hemodynamics changes affect kidney function and may eventually lead to chronic kidney disease. How might kidney function decline differ in male and female patients with diabetes, and why?

A related issue concerns the anti-hyperglycemic drugs that target the kidney. As mentioned above, the kidney plays a major role in homeostasis, including the regulation of blood glucose levels (Shepard, 2019). Following glomerular filtration, almost all glucose is reabsorbed from the lumen of the kidney within the proximal tubule, *via* two major Na^+^-glucose cotransporters (SGLT1 and SGLT2), such that there is no loss of glucose. When insulin production is reduced, blood glucose levels increase and the task of handling this excess glucose load falls on the kidney. In patients with diabetes, filtered blood glucose levels exceed the transport capacity of SGLT1 and SGLT2, leading to the excretion of glucose in urine, i.e., glycosuria. Historically, glycosuria has been associated with diabetes, but with the prescription of SGLT inhibitors, this pathophysiological condition is now used as a mechanism to lower blood glucose (Chao and Henry, 2010; Carpentier et al., 2019). Specifically, SGLT2 inhibitors block the glucose reabsorption along the early proximal tubule and induce glycosuria to reduce blood glucose levels. Since SGLT2 mediates the co-transport of glucose and Na^+^, inhibition of SGLT2 induces natriuresis and diuresis upon reduction of Na^+^ and fluid reabsorption in the proximal tubule. Thus, besides their intended anti-hyperglycemic effect, SGLT2 inhibitors have been shown to lower blood pressure and provide protection from heart failure (Zinman et al., 2015; Neal et al., 2017). Given the promises of SGLT2 inhibitors, it seems imperative to understand thoroughly their mechanisms of action, some of which have remained unclear, as well as any sex-dependent kidney response to these drugs.

We have previously conducted model simulations to investigate kidney function in diabetes and the renal effects of SGLT2 inhibition on the kidney of a male rat or human (Layton et al., 2015, 2016a; Layton and Vallon, 2018; Hu and Layton, 2021). As such, even though some of the model predictions may generalize to women, the translational value of any study that involves only half of the population remains limited. Thus, the goal of the present study is to develop sex-specific models that allow us to analyze and compare kidney function in male and female patients with different stages of diabetes, and the renal effects of SGLT2 inhibition.

## Materials and Methods

**FIGURE 1:**
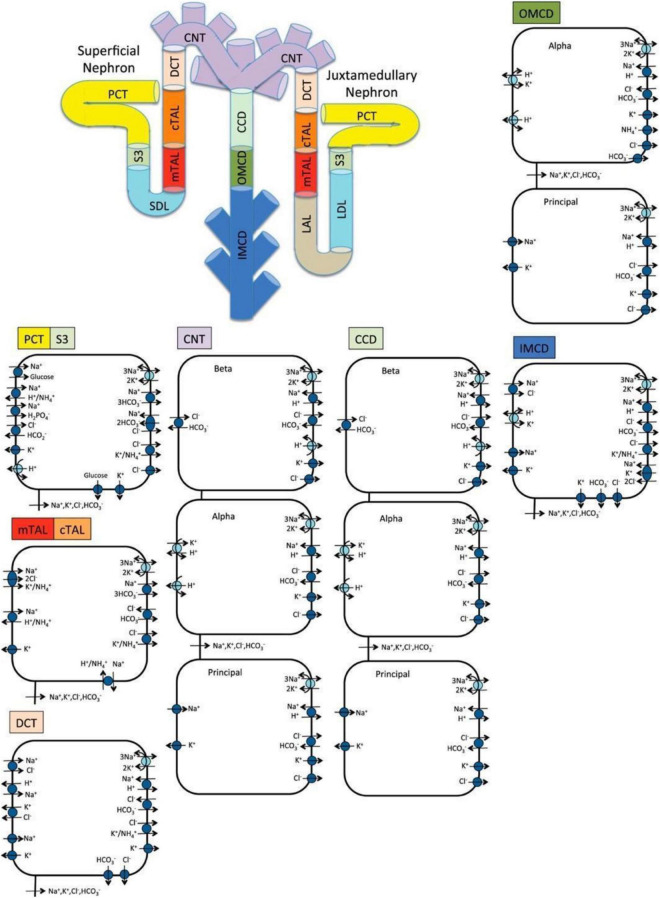
Schematic diagram of the nephron system (not to scale). The model includes 1 representative superficial nephron and 5 representative juxtamedullary nephrons, each scaled by the appropriate population ratio. Only the superficial nephron and one juxtamedullary nephron are shown. Along each nephron, the model accounts for the transport of water and 15 solutes (see text). The diagram displays only the main Na^+^, K^+^, and Cl^–^ transporters. mTAL, medullary thick ascending limb; cTAL, cortical thick ascending limb; DCT, distal convoluted tubule; PCT, proximal convoluted tubule; CNT, connecting duct; CCD, cortical collecting duct; SDL, short or outer-medullary descending limb; LDL/LAL, thin descending/ascending limb; OMCD, outer-medullary collecting duct; IMCD, inner-medullary collecting duct.

We previously implemented an epithelial cell-based model of transporter-mediated solute transport along the nephrons of a human kidney (Layton and Layton, 2019; Hu et al., 2021); that model was recently extended to simulate kidney function in a male patient with diabetes (Hu and Layton, 2021). In this study, we extend that model to simulate kidney function in a female patient with diabetes. The model represents the following six classes of nephrons: a superficial nephron, which turns at the outer-inner medullary boundary, and five juxtamedullary nephrons, of different lengths, each reaching into differing levels of the inner medulla. While the superficial nephrons account for 85% of the nephron population, and extend from the Bowman’s capsule to the papillary tip, the remaining 15% of the nephrons are juxtamedullary that possess loops of Henle that reach to different depths in the inner medulla; most of the long loops turn within the upper inner medulla. Population-based weighted average is taken wherever necessary. Each model nephron is represented as a tubule with a variety of transporters on the apical and basolateral membrane. The model assumes that ten connecting tubules coalesce successively to one cortical collecting tubule (Oliver, 1968). In the inner medulla, the collecting ducts again coalesce successively in the ratio of 10:1. A schematic diagram for the model is shown in Figure 1.

The model accounts for the following 15 solutes: Na^+^, K^+^, Cl^–^, HCO_3_^–^, H_2_CO_3_, CO_2_, NH_3_, NH_4_^+^, H_2_PO_4_^–^, H^+^, HCO_2_^–^, H_2_CO_2_, urea, and glucose. The model is formulated for steady state and consists of a large system of coupled ordinary differential equations and algebraic equations (Layton and Layton, 2019). Model solution describes luminal fluid flow, luminal fluid solute concentrations, and hydrostatic pressure. Excluding the descending limb segment, model solution also describes cytosolic solute concentrations, membrane potential, and transcellular and paracellular fluxes. In a non-diabetic kidney, we assume a single-nephron glomerular filtration rate (SNGFR) of 100 and 133 nl/min for the superficial and juxtamedullary nephrons, respectively, in both women and men. Assuming a total of 1 million nephrons, this yields a GFR of 105 mL/min. Model parameters that describe a non-diabetic human kidney can be found in Layton and Layton (2019).

### Sodium-Glucose Cotransport in the Proximal Tubule

Under euglycemic conditions, the SGLTs facilitate the reabsorption of most of the filtered load of glucose, working in tandem with the glucose transport facilitators (GLUT) on the basolateral side of the proximal tubule cells. The proximal convoluted tubule expresses the high-capacity, low-affinity transporter SGLT2 and GLUT2 and the S3 segment expresses the lower capacity, higher affinity transporter SGLT1 and GLUT1. The modeling of glucose and Na^+^ fluxes across SGLT2 and SGLT1 cotransporters, and glucose fluxes across GLUT1 and GLUT2 have been described in our previous studies (Layton et al., 2015, 2016a,2016b,2016c).

### Simulating Sex-Specific Kidney Function

We have previously developed sex-specific models of kidney function for the rat (Hu et al., 2019, 2020) and for humans (Hu et al., 2021). To produce those models, we formulated epithelial cell-based models of solute transport along male and female rat nephrons. First, sex-specific epithelial transport models were formulated only for the proximal tubule, then for the thick ascending limb, for the distal convoluted tubule, for the connecting tubule, and then individually for the cortical and medullary segments of the collecting ducts. The male and female transport models account for sex differences *via* the expression levels of apical and basolateral transporters (Sabolić et al., 2012; Veiras et al., 2017); which also vary among nephron segments. The water permeability is lowered by 36% in the proximal convoluted tubules on the basolateral end and doubled in the distal tubules and collecting ducts in women compared to men. Sodium and chloride permeability is lowered by 50 and 60% and in PCT and S3 in women. Due to the high Na distal capacity seen in women, Na permeability was increased by 40%. Activity of sodium transporters such as NKCC2 and NaKATPase was increased by 20% in the ascending limbs, distal tubules and connecting ducts in women. Nephron segment lengths and luminal diameters in the human kidney are taken to be the same in women and men due to the absence of sex-specific human data; additional model parameters can be found in Layton and Layton (2019). Key differences in nephron transport parameters between non-diabetic women and men are summarized in Supplementary Table 1 with significantly different ones marked in red.

### Simulating a Diabetic Kidney

Diabetes is associated with renal hypertrophy, hyperfiltration, and alterations in transporter expression (Nauck, 2014; Bjornstad and Cherney, 2018; Lovshin et al., 2018; Shepard, 2019). In this study, we simulate two diabetic conditions:

(1) Moderate diabetes: plasma glucose is elevated from its non-diabetic value of 5–8.6 mM; SNGFR is increased by 27% (Mogensen, 1971); the tubular diameter and length of the proximal tubules are increased by 10%; and the diameter and length of the distal segments are increased by 18 and 7% (Baumgartl et al., 1998; Layton et al., 2016a);

(2) Severe diabetes: plasma glucose is further increased to 20 mM; SNGFR is increased by 10% (Mogensen, 1971); the tubular diameter and length of the proximal tubules are increased by 28%; the diameter and length of the distal segments are increased by 42 and 7%, respectively (Baumgartl et al., 1998; Layton et al., 2016a).

Due to lack of sex-specific data, we assume the same morphological changes in both men and women and a similar enhancement in transcellular water permeability along the cortical and inner-medullary collecting duct segments by 55 and 40%, respectively, in moderate and severe diabetes (Layton et al., 2016a). The altered transporter activities are summarized in Table 1.

**TABLE 1:** Up- (↑) or downregulation (↓) of transporter activity in moderate and severe diabetes.

Transporter type	Segment	Moderate diabetes		Severe diabetes	
		Female	Male	Female	Male
SGLT2	PCT/S3	38%↑	38%↑	28%↑	38%↑
SGLT1	PCT/S3	33%↓	33%↓	33%↓	33%↓
GLUT2	PCT/S3	50%↑	30%↑	30%↑	50%↑
Na^+^–K^+^–ATPase	PCT/S3	10%↑	10%↑	10%↑	10%↑
	mTAL	20%↑	20%↑	10%↑	20%↑
	cTAL, DCT, CNT, CCD, OMCD	10%↑	10%↑	5%↑	10%↑
	IMCD	150%↑	150%*^a^*↑	50%*^a^*↑	150%↑
NKCC2	mTAL	10%↑	10%↑	5%↑	10%↑

*PCT, proximal convoluted tubule; mTAL/cTAL, medullary/cortical thick ascending limb; DCT, distal convoluted tubule; CNT, connecting tubule; CCD/OMCD/IMCD, cortical/outer-medullary/inner-medullary collecting duct.*

*^a^50% along the first 2/3rd of the segment and 150% along the rest.*

### Simulating SGLT2 Inhibition

We assume that following acute SGLT2 inhibition, SNGFR decreases by 3% in all nephrons, in accordance to the minor 3% GFR reduction seen in non-diabetic subjects, being administered canagliflozin or dapagliflozin for 4 days (Layton et al., 2016a). Also, in non-diabetic subjects, SGLT2 inhibition yields a higher urinary excretion of glucose (45% of filtered load) (Seman et al., 2013). In our previous study, we modeled 90% inhibition of SGLT2 in all nephrons, resulting in the excretion of 40% of the filtered glucose, to approximate this glucose excretion (Seman et al., 2013). Having established the effect of SGLT2 inhibition in healthy patients, it is seen that SGLT2 inhibition attenuates diabetic-reduced hyperfiltration in diabetic patients (Cherney et al., 2014). Thus, when simulating the effects of SGLT2 blockade in a diabetic kidney, we lower GFR to its non-diabetic level of 105 ml/min. Upon acute administration, SGLT2 inhibition is assumed not to affect plasma glucose concentration; thus, blood glucose level is kept at 8.6 and 20 mM in the moderate and severe diabetes cases, respectively.

## Results

### Kidney Function Under Diabetic Conditions

**FIGURE 2:**
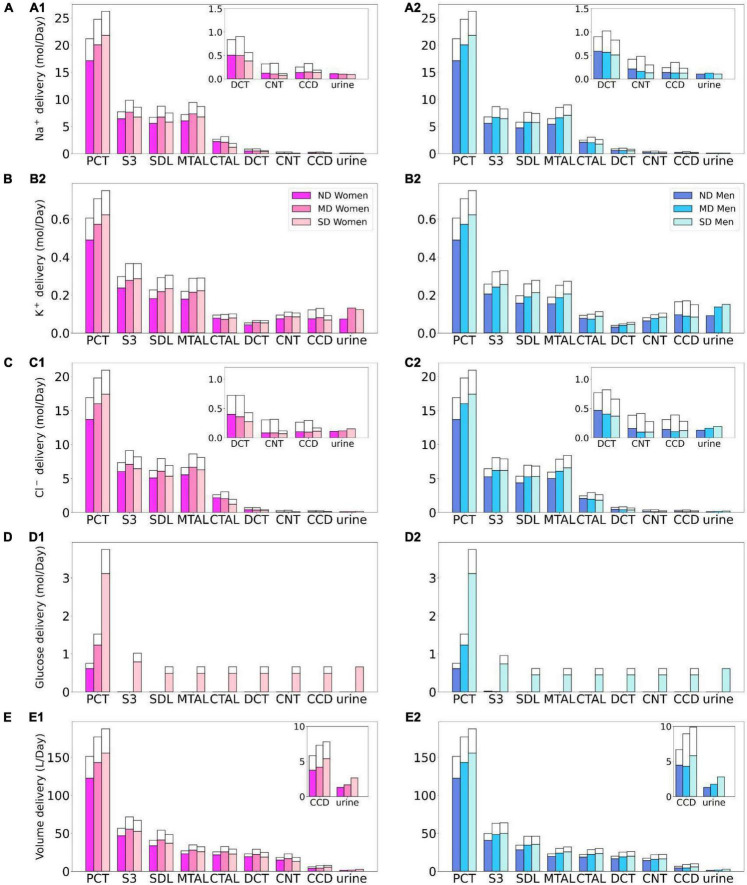
Delivery of Na^+^ **(A)**, K^+^ **(B)**, Cl^–^ **(C)**, glucose **(D)**, and fluid **(E)** to the beginning of individual nephron segments in women and men without diabetes (ND) and with moderate (MD) and severe diabetes (SD). Color bars, superficial nephron values; white bars, juxtamedullary values, computed as weighted totals of the five representative model juxtamedullary nephrons. The models assume that superficial nephrons account for 85% of the nephron population; thus, the superficial delivery values are substantially higher. In each case, only one bar is shown for “urine” since the superficial and juxtamedullary nephrons have merged at the cortical collecting duct entrance. PCT, proximal convoluted tubule; SDL, descending limb; MTAL/CTAL, medullary/cortical thick ascending limb; DCT, distal convoluted tubule; CNT, connecting tubule; CCD, cortical collecting duct.

**FIGURE 3:**
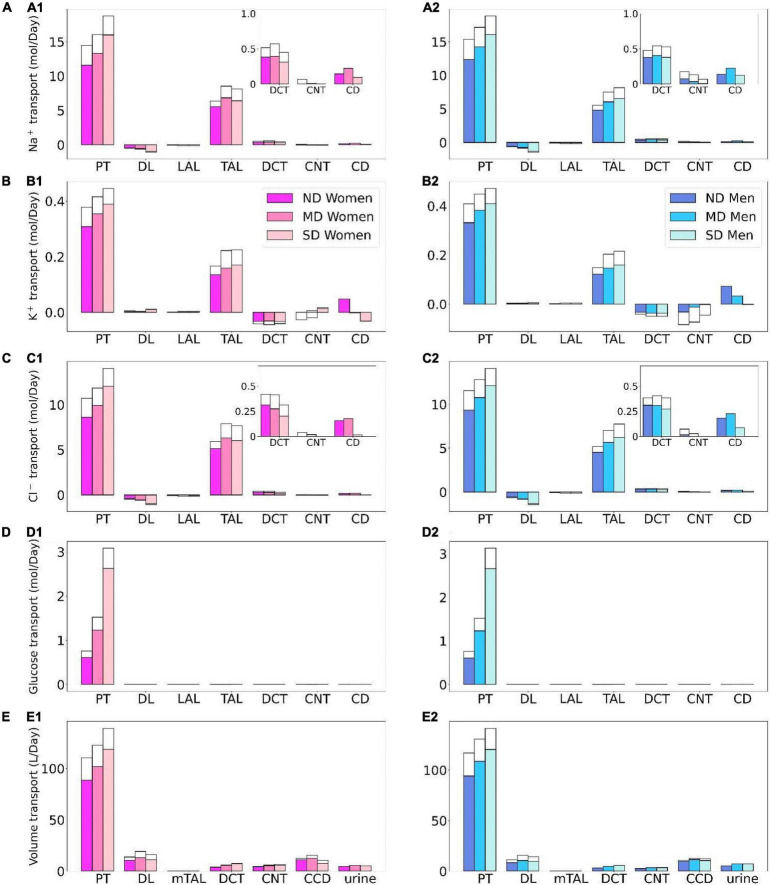
Transport of Na^+^ **(A)**, K^+^ **(B)**, Cl^–^ **(C)**, glucose **(D)**, and fluid **(E)** along individual nephron segments in women and men without ND, with MD, and with SD. Notations are analogous to Figure 2. PT, proximal convoluted tubule and S3.

**FIGURE 4:**
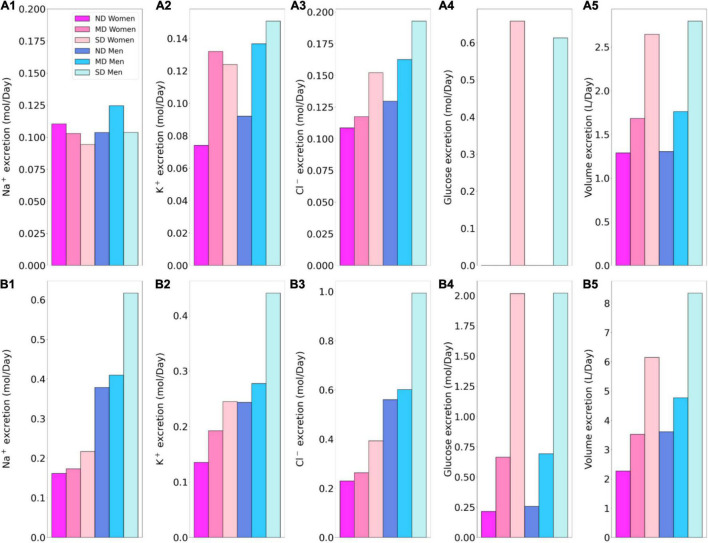
Total excretion of Na^+^ (*1*), K^+^ (*2*), Cl^–^ (*3*), glucose (*4*) and fluid (*5*) in men and women without ND, with MD, and with SD. Results are obtained under no inhibition **(A1–A5)** and with SGLT2 inhibition **(B1–B5)**.

We investigate the change in solute and water transport along the nephrons due to diabetes, and if those changes differ between men and women. Key results are summarized in Figures 2–4. In these simulations, we mimic the renal effects of moderate and severe diabetes as described in the “Materials and Methods” section. In particular, diabetes induces glomerular hyperfiltration and tubular hypertrophy, to different extents depending on the severity of the disease. Elevated GFR is reflected on filtered solute loads, whereas tubular hypertrophy results in enhanced transport, as discussed below. We will first compare nephron transport in a non-diabetic and diabetic kidney between the two sexes. Then we will dive deeper into the sex differences.

The proximal tubules in a healthy kidney reabsorb essentially all filtered glucose. In a healthy kidney, the proximal convoluted tubule in both women and men reabsorbs 97% of filtered glucose *via* the SGLT2, and the S3 segments reabsorb almost all of the remaining glucose *via* the SGLT1 (see Figure 2D). In a diabetic kidney, the filtered glucose load increases substantially, but so does the glucose transport capacity. In the moderate diabetes simulations, plasma glucose concentration is assumed to increase from 5 to 8.6 mM, which together with the elevated GFR yields a filtered load of 1.52 mol⋅day^–1^ glucose, which is twice the regular filtered load. However, as the proximal tubules hypertrophize under diabetic conditions, they gain 10% in both length and diameter, which corresponds to ∼20% increase in effective transport area. Thus, the enhanced proximal tubule glucose transport balances the higher filtered load, resulting in the absence of glycosuria (glucose excretion in urine) in patients with moderate diabetes (Nauck, 2014).

In severe diabetes simulations, plasma glucose is further elevated to 20 mM, resulting in glucose filtered load of 3.75 mol⋅day^–1^ glucose. The length and diameter of the proximal tubules further increase to 28% above baseline, resulting in a 64% increase in effective transport area. Despite its enhanced transport capacity, the glucose load exceeds the transport capability of the proximal tubule. Net glucose reabsorption by the proximal convoluted tubule and S3 segment increases, but because of the increased filtered load, fractional reabsorption along the proximal convoluted tubule is predicted to decrease to 72.7% in women and 74.5% in men, whereas the S3 accounts for 0.1% of the filtered glucose in both sexes. Because downstream segments do not possess significant glucose transport capacities, the severe diabetic model predicts female and male glucose excretion of 0.65 and 0.61 mol⋅day^–1^, respectively.

Besides glucose, diabetes affects renal transport of electrolytes and water as well. The 10% increase in GFR in the moderate diabetic kidney, relative to a healthy kidney, implies a 10% increase in filtered Na^+^ and also ∼10% increase in total Na^+^ transport, since only ∼1% of the filtered Na^+^ is excreted. The model predicts that the increased transport activities occur primarily by the tubular segments where the Na^+^ transporter activities are significantly elevated in diabetes (Figure 3A). In particular in the proximal convoluted tubules the hyperfiltration-induced changes in the torque augment the density of all transcellular transporters, and in the medullary thick ascending limbs diabetes enhances the density of NKCC2 (Layton et al., 2016a). Then, enhanced Na^+^ transport can compensate for the elevated filtered Na^+^ load in diabetes, resulting in Na^+^ excretion comparable to a non-diabetic kidney; see Figures 2A, 4A1. Due to the coupled action of NKCC2, elevated Na^+^ reabsorption is followed by increases in the reabsorption of Cl^–^ (Figures 2C,E; Seman et al., 2013). Urine output is predicted to be 30 and 35% higher in moderately diabetic women and men, respectively, due to osmotic diuresis (Figures 2E, 4A5). Higher filtered K^+^ load enhances its tubular reabsorption, similar to Na^+^, along the proximal tubules and thick ascending limbs (Figure 2B). These competing factors result in kaliuresis, with K^+^ excretion predicted to be 78 and 48% higher in women and men with moderate diabetes, respectively (Figures 2B, 4A2).

Severe diabetes induces GFR and filtered solute loads by 24% above baseline. The resulting changes in tubular transport are similar to moderate diabetes. In both women and men, the enhanced Na^+^ transport along the proximal tubules and thick ascending limbs essentially compensates for the elevated filtered Na^+^ load in diabetes to limit natriuresis (Figures 2A, 4A1). Osmotic diuresis approximately doubles urine output (Figures 2E, 4A5), and urinary K^+^ excretion is approximately two-third higher (Figures 2B, 4A2).

In sum, diabetes markedly increases GFR and filtered solute loads. That is accompanied by tubular hypertrophy and enhanced renal transport capacity, which prevents excessive loss in fluid and electrolytes (Figure 4A). The model predicts that the kidneys of women and men with diabetes handle water, electrolytes, and glucose in manners that are qualitatively similar. In the next set of simulations, we examine sex differences in kidney function with SGLT2 inhibition.

### Kidney Function in Non-diabetic Women and Men Under SGLT2 Inhibition

**FIGURE 5:**
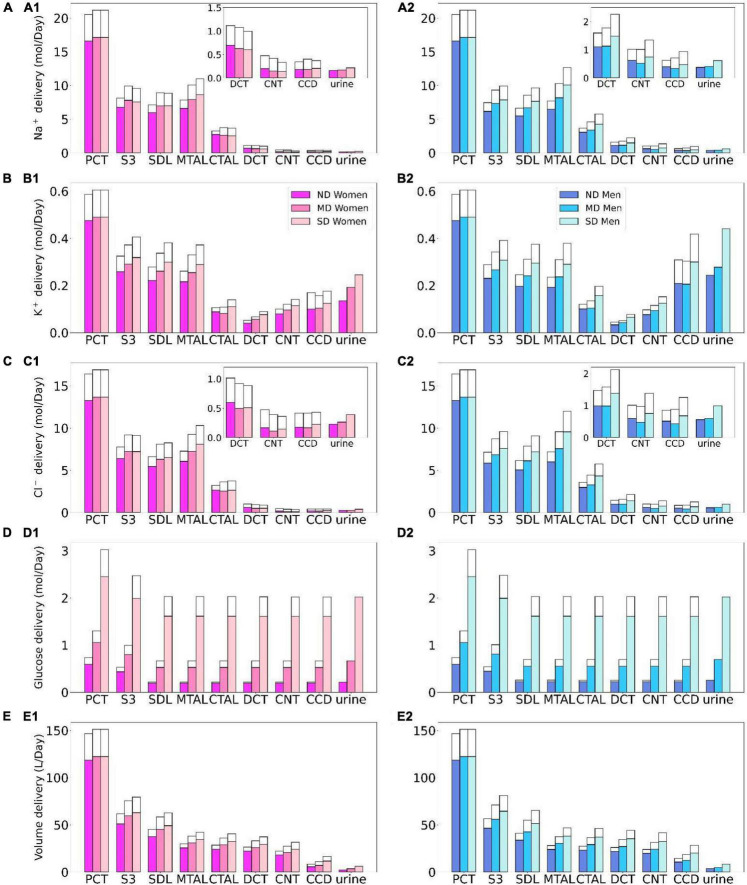
Delivery of Na^+^ **(A)**, K^+^ **(B)**, Cl^–^ **(C)**, glucose **(D)**, and fluid **(E)** to the beginning of individual nephron segments in women and men without ND, with MD, and with SD under SGLT2 inhibition. Notations are analogous to Figure 2.

The model simulates the administration of a SGLT2 blockade by inhibiting 90% of SGLT2. In a non-diabetic kidney, the remaining 10% of the SGLT2 mediates the reabsorption of 28 and 26% of the filtered glucose along the proximal convoluted tubules in women and men, respectively. The much-elevated glucose flow into the S3 segments increases that segment’s SGLT1-mediated glucose transport to 39 and 43% of filtered load in women and men, respectively. Due to its osmotic diuretic effect of the SGLT2 inhibition (Layton et al., 2015, 2016a), passive paracellular reabsorption is attenuated in the proximal tubule. The higher luminal flow stimulates active transport (*via* torque-induced increases in transcellular transporter expression (Layton et al., 2017), but the reduction in passive transport dominates. As a result, net Na^+^ reabsorption decreases in the proximal tubules, by 9.1 and 7.9% in women and men, respectively. Solute transport shifts downstream, primarily to the medullary thick ascending limbs, but also to the distal segments (Figure 5A). The increase in thick ascending limb Na^+^ transport is larger in women compared to men, resulting in more severe natriuresis in men, who exhibit a 265% increase in Na^+^ excretion with SGLT2 inhibition, compared to a 46% increase in women. The elevated Na^+^ reabsorption along the connecting tubules is accompanied by enhanced K^+^ secretion (Figure 5B). Despite the enhanced transport, SGLT2 inhibition in a non-diabetic kidney induces diuresis and kaliuresis. Urine output increases by 76% in women and to an even larger extent in men by 177%. Similarly, K^+^ excretion increases by 82 and 164% in women and men, respectively (Figures 4B1–B5).

Taken together, SGLT2 inhibition not only induces glycosuria, but also diuresis (Figure 5E), natriuresis, and kaliuresis in non-diabetic women and men. Unlike the earlier results without SGLT2 inhibition, these SGLT2 inhibition results show significant sex differences. The effects on urine excretion are stronger in men, in part due to the larger Na^+^ transport capacity of the thick ascending limbs in the kidneys of women, which allow those segments to compensate for the reduction in transport upstream. Note that there is no evidence of significant diuresis or kaliuresis in clinical trials (Whaley et al., 2012; Kapur et al., 2013; Seman et al., 2013; Sha et al., 2014; Zinman et al., 2015; Neal et al., 2017; Eickhoff et al., 2019; McMurray et al., 2019). In the absence of a renin-angiotensin system in the model, it is possible that the model does not reach the correct electrolyte and water balance, which may explain the discrepancy in model prediction and experimental evidence.

### Kidney Function in Diabetic Women and Men Under SGLT2 Inhibition

Kidney function under SGLT2 inhibition in a patient with diabetes is arguably the most clinically relevant case. Recall that SGLT2 inhibition attenuates diabetes-induced glomerular hyperfiltration and returns GFR to baseline, lowering the filtered glucose load from 1.52 to 1.3 mol⋅day^–1^ in moderate diabetes. The kidney’s response in glucose transport is similar in women and men: proximal convoluted tubule glucose reabsorption, mediated by the 10% remaining SGLT2, reduces by ∼80%, from 1.5 to 0.3 mol glucose⋅day^–1^ (Figure 5D). The SGLT1 along the S3 segment reabsorbs a fraction of the remaining glucose, at a rate similar to the proximal convoluted tubule, thereby limiting the risk of hypoglycemia (Nespoux and Vallon, 2018). Glucose excretion in moderately diabetic women and men is predicted to be similar, at 0.69 and 0.66 mol⋅day^–1^, respectively, which corresponds to almost 50% of filtered glucose; see Figure 4B4.

**FIGURE 6:**
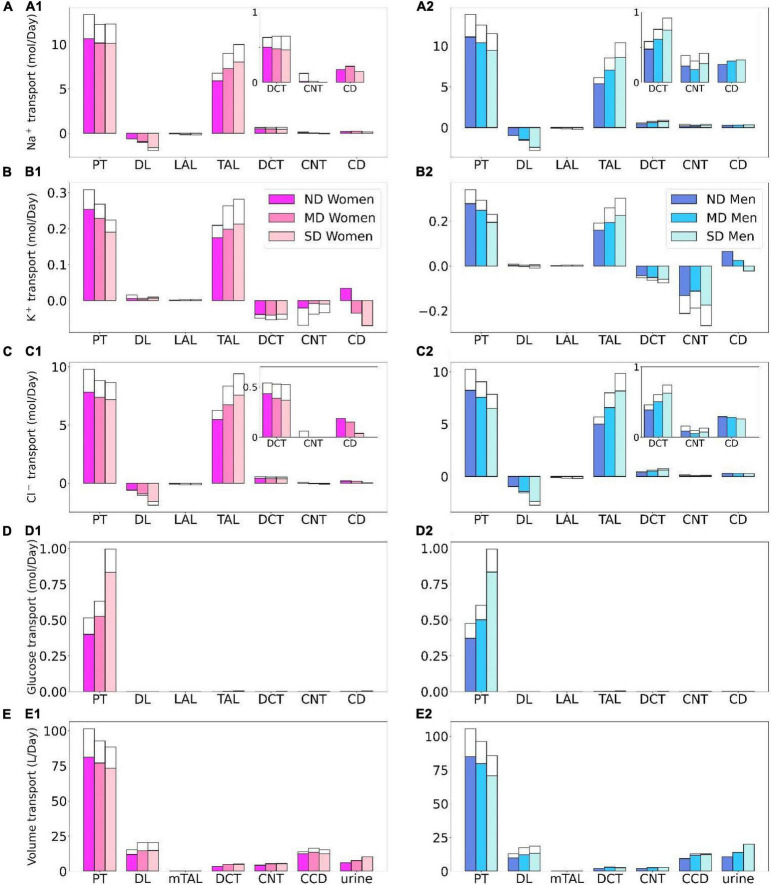
Transport of Na^+^ **(A)**, K^+^ **(B)**, Cl^–^ **(C)**, glucose **(D)**, and fluid **(E)** along individual nephron segments in women and men without ND, with MD, and with SD under SGLT2 inhibition. Notations are analogous to Figure 2. PT, proximal convoluted tubule and S3.

The kidney’s response to SGLT2 inhibition in severe diabetes exhibits similarities but also notable differences from the moderate diabetes case. Even though SGLT2 inhibition normalizes GFR, the plasma glucose level remains high at 20 mM, which yields a filtered glucose load of 3.0 mol glucose⋅day^–1^. Proximal tubule glucose transport is similar in women and men with severe diabetes, with the proximal convoluted tubule and S3 reabsorbing ∼0.55 and 0.45 mol glucose⋅day^–1^, respectively (Figure 6D). Together, these two segments account for two-third of the filtered glucose. The predicted glucose excretion of 2.02 mol⋅day^–1^ in both sexes (Figure 4B4) is consistent with reported values (List and Whaley, 2011). A major difference between the moderate and severe diabetes cases lies in the S3 response. Recall that in moderate diabetes, glucose transport along the S3 segment jumps from negligible to ∼0.3 mol glucose day^–1^, which corresponds to 23% of filtered glucose following SGLT2 inhibition. In contrast, in severe diabetes, even without SGLT2 inhibition, S3 already mediates the reabsorption of a significant fraction of the filtered glucose (0.36 mol glucose day^–1^ or 9% of the filtered glucose), because the glucose transport capacity of the proximal convoluted tubule is overwhelmed. Following SGLT2 inhibition, the increase in S3 glucose transport is less drastic, specifically, an increase of 25% to 0.45 mol glucose day^–1^ (compare Figures 4A4,B4). In addition, whereas there is glucose secretion across the tight junction of the proximal convoluted tubules in the absence of treatment, the paracellular pathway mediates glucose reabsorption when SGLT2 is inhibited (owing to higher luminal glucose concentration).

While the primary target of SGLT2 inhibitors is glucose, they also have a significant impact on renal Na^+^ transport. By normalizing GFR, SGLT2 inhibition significantly lowers filtered Na^+^ load and Na^+^ transport. Our simulations suggest that SGLT2 inhibition induces osmotic diuresis in the proximal tubules, as in the non-diabetic case, thereby reducing paracellular transport. In fact, the model predicts that osmotic diuresis reverses the direction of paracellular Na^+^ transport in S3: the luminal-to-interstitial Na^+^ concentration gradient favors Na^+^ secretion into the lumen *via* the tight junctions. Consequently, in the moderate diabetes case, Na^+^ excretion increases by 68 and 228% in women and men, respectively (Figure 4B1). Natriuresis is less severe in women because the thick ascending limbs in their kidneys are more capable of compensating for the reduced Na^+^ transport by the proximal convoluted tubules. Importantly, natriuresis is also accompanied by diuresis, with urine output increases of 109 and 170%, in women and men, respectively. The elevated Na^+^ along the distal tubular segments raises K^+^ secretion. In men, that yields an increase of 103% in K^+^ excretion compared to the cases without SGLT2 inhibition (Figure 4B2). In women, the distal tubular Na^+^ flow is lower than in men, as is K^+^ secretion. As such, kaliuresis is attenuated in women, with K^+^ excretion 45% higher than without SGLT2 inhibition. Qualitatively similar results are obtained for the severe diabetes case; see Figures 4B1, 5A, 6A.

Taken together our results indicate that the intended glycosuric effect of SGLT2 inhibitors is similar in women and men with diabetes. SGLT2 inhibition also induces diuresis, natriuresis, and kaliuresis, to a more severe extent in men compared to women.

### The Influence of Sex and SGLT2 Inhibition on the Tubuloglomerular Feedback Signal

At the onset of diabetes, the kidney hypertrophies and SNGFR increases. A causal link between the two pathophysiological processes has been proposed, based on the tubuloglomerular feedback. The tubuloglomerular feedback adjusts SNGFR based on the luminal [Cl^–^] sensed by the macula densa cells: if the [Cl^–^] exceeds a target value, the tubuloglomerular feedback is activated, and a signal is sent to constrict the afferent arteriole and reduce SNGFR, and vice versa (Layton, 2010). The tubuloglomerular feedback is believed to play a role in the diabetes-induced glomerular hyperfiltration: the enhanced proximal tubular transport lowers the tubuloglomerular feedback signal, i.e., the luminal [Cl^–^] sensed by the macula densa cells, resulting in a feedback-induced increase in SNGFR (Sgouralis and Layton, 2014). Because the tubuloglomerular feedback is not characterized in humans, it is not explicitly represented in the present models. Instead, SNGFR is assumed to be known *a priori*. Nevertheless, we present here our model results to assess the potential influences of sex and SGLT2 inhibition on the tubuloglomerular feedback, and the implications on SNGFR.

**FIGURE 7:**
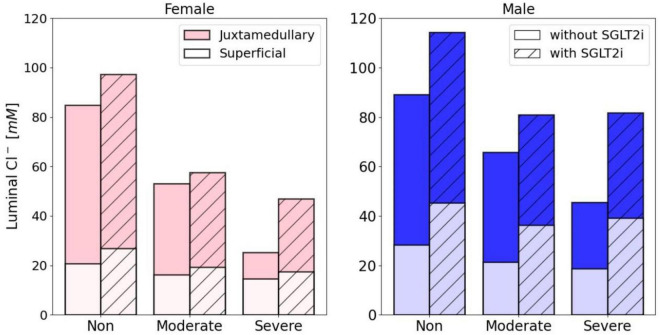
Luminal [Cl^–^] (mM) at the macula densa for non, moderately and severely diabetic women and men. The contribution from one superficial nephron and five juxtamedullary nephrons are both shown here. To calculate an average value, we would have to take a population weighted average of 85: 15 superficial: juxtamedullary. Note that the juxtamedullary values span the full y-value. For example, for non-diabetic women under no SGLT2 inhibition, the contribution from the juxtamedullary nephron is 84.9 mM.

Figure 7 shows predicted luminal [Cl^–^] at the macula densa, where the sensing of the tubuloglomerular feedback signal occurs and is taken to be the end of the cortical thick ascending limbs. Values are computed for the superficial nephron, and as weighted averages of the juxtamedullary nephrons. For both women and men, diabetes lowers macula densa [Cl^–^] (Figures 3C, 5C), which would attenuate the tubuloglomerular feedback signal and would lead to glomerular hyperfiltration. The macula densa [Cl^–^] is predicted to be lower in women in all cases. Taken in isolation, a lower macula densa [Cl^–^] should correspond to higher SNGFR. In contrast, after correction for weight, GFR is not known to differ significantly between sexes (Jacobsen et al., 1999; de Hauteclocque Ragot et al., 2014). Thus, the kidneys of women and men may have different tubuloglomerular feedback operating points. That is, given different baseline macula densa [Cl^–^] values, the different tubuloglomerular feedback systems in women and men would generate similar SNGFR.

The model predictions also indicate a GFR-normalizing effect of SGLT2 inhibition. By limiting Na^+^-glucose transport, SGLT2 inhibition increases macula densa [Cl^–^] substantially (Figure 7), which would activate the tubuloglomerular feedback and lower SNGFR. It is noteworthy that the increases in macula densa [Cl^–^] are significantly larger in men than in women. Taken in isolation, that might suggest that SGLT2 inhibition may be more effective in ameliorating diabetes-induced glomerular hyperfiltration in men compared to women. However, such sex difference has not been reported, a discrepancy that may be attributed to the involvement of factors not represented in the model (see “Discussion”).

### Sensitivity Analysis

To assess the sensitivity of model behaviors to changes in key parameters, we conducted a sensitivity analysis, in which we varied the activities of glucose transporters (SGLT2, SGLT1, GLUT2; Veiras et al., 2017) and key Na^+^ transporters NKCC2 (Musselman et al., 2010) and Na^+^-K^+^-ATPase (Fraser and Sarnacki, 1989) that are known to be major determinants of electrolyte excretions and exhibit sex differences. Given the substantial uncertainties in these transporter activities, we varied the corresponding activities individually by ±20% in all segments and determined the effect on urine output, glucose and electrolyte excretion rates. The sensitivity study was conducted in both men and women, under healthy, moderately and severely diabetic conditions, all with SGLT2 inhibition.

**FIGURE 8:**
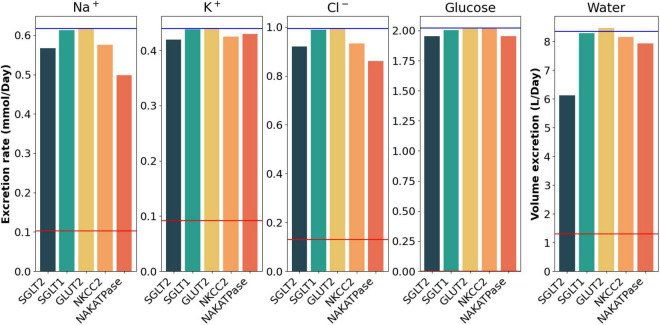
Urinary excretion of Na^+^, K^+^, Cl^–^, glucose, and fluid, computed for severely diabetic men. Results are obtained with SGLT2 inhibition upon a 20% increase in the individual activity of the five specific transporters: SGLT2, SGLT1, GLUT2, NKCC2, Na^+^-K^+^-ATPase. The red lines denote the solute and volume prediction in healthy male under no drugs. The blue lines indicate the levels predicted by the model for severely diabetic males under 90% SGLT2 inhibition.

Figure 8 shows results for the male model with severe diabetes, with a 20% increase in individual transporter activities. For SGLT2, that 20% was computed relative to its inhibition level. Results for female and other conditions are qualitatively similar. As expected, we observe a reduction in urine volume and solute excretion upon a 20% increase in SGLT2, NKCC2, and Na^+^-K^+^-ATPase activities (compare bars with blue lines). The predicted decreases in urinary volume and excretion are notably less than the 20% increase in transporter activities and remain well above the control values (no SGLT2 inhibition, red lines). Results for female models and male model with moderate diabetes are added in the Supplementary Material 1. These results indicate the robustness of the system.

## Discussion

In addition to regulating fluid, electrolytes, and blood pressure, the kidney also plays a key role in glucose homeostasis. In the euglycemic state, essentially all the filtered glucose is reabsorbed by the proximal tubules *via* the SGLT2 and SGLT1, with little glucose excreted in urine. In diabetes, where either insulin production or sensitivity is impaired, the blood glucose level rises. Chronic exposure to elevated blood glucose levels contribute to the tubular hypertrophy observed in diabetes. In turn, this increase in epithelial cell area due to tubular hypertrophy increases the transport capacity, which may increase SNGFR by suppressing tubuloglomerular feedback (more below). The activities of SGLT2/SGLT1 are likely modified in patients with diabetes; however, those changes have not been characterized. Nonetheless, when sufficiently high plasma glucose is coupled with glomerular hyperfiltration, the kidney’s glucose transport capacity may be overwhelmed, resulting in the appearance of glucose in urine, which is traditionally considered a hallmark of diabetes. In fact, the Chinese translation of “diabetes” is literally “sugar in urine” disease.

The model findings presented here have provided insights into the mechanisms that give rise to glomerular hyperfiltration in early diabetes, its normalization by SGLT2 inhibitors, and potential sex differences. Consistent with the “tubulo-centric” hypothesis by Vallon and co-workers, predictions by both the models for women and men indicate that the enhanced reabsorption along the proximal tubules in early diabetes lowers the luminal [Cl^–^] sensed by the macula densa cells, suppresses the tubuloglomerular feedback signal, and results in a feedback-induced increase in SNGFR. These model findings are consistent with micropuncture findings in rats (Vallon et al., 1999; Thomson and Vallon, 2021). It must be noted that the presence of a tubuloglomerular feedback has yet to be established in the human kidney. Nonetheless, if tubuloglomerular feedback is present in humans, the vasodilative signal predicted in diabetes could contribute to glomerular hyperfiltration. In addition, if humans exhibit the sexual dimorphism in renal transporter pattern as observed in rodents, then the tubuloglomerular feedback signal likely differs between women and men. For women and men to have similar SNGFR, the operating points of their tubuloglomerular feedback systems may have to be adjusted accordingly, to map different macula densa [Cl^–^] values to the same SNGFR.

Moreover, the model predicts that SGLT2 inhibition while lowering Na^+^ and glucose transport, significantly increases luminal [Cl^–^] at the macula densa (Figure 7), which may then attenuate glomerular hyperfiltration *via* tubuloglomerular feedback, consistent with clinical data, which show a reduction in eGFR in patients with diabetes following chronic SGLT2 inhibition. The model predicts that SGLT2 inhibition increases macula densa luminal [Cl^–^] to a larger extent in men than in women. However, such sex difference in drug response has not been reported. That discrepancy may be attributed to factors such as the resetting of tubuloglomerular feedback (i.e., adjustment of its operating point) (Thomson and Blantz, 2008), or sex-specific transporter alterations in diabetes and SGLT2 inhibition that have yet to be characterized. Nevertheless, for both women and men, lowering glomerular hyperfiltration on the single nephron level by SGLT2 inhibition may provide long-term beneficial effects in the diabetic kidney, by reducing the transport load and metabolic requirement on the nephrons (Ferrannini and Solini, 2012; Neal et al., 2017).

In sum, we have developed the sex-specific computational models of detailed epithelial transport in the kidney of a patient with diabetes. The models predict that, similar to rodents, diabetes-induced tubular hypertrophy in both men and women may contribute to glomerular hyperfiltration *via* a (hypothesized) tubuloglomerular feedback signal. Model predictions further suggest that SGLT2 inhibition may activate tubuloglomerular feedback and attenuate glomerular hyperfiltration, and the tubuloglomerular feedback signal may be stronger in men. Model simulations did not reveal significant differences in renal handling of electrolyte and water handling between women and men with diabetes. More notable sex differences emerge in simulations of SGLT2 inhibition, which induces diuresis, natriuresis, and kaliuresis, with those effects significantly more prominent in men compared to women.

Although there is no major sex-specific differences seen in the larger clinical trials of SGLT2 inhibitors, we still find comparability in our predictions of increase in solute excretion with some trials (Sha et al., 2014; Masuda et al., 2020; van Bommel et al., 2020) and not so much with others (Kapur et al., 2013; Cherney et al., 2014; Tanaka et al., 2017; Eickhoff et al., 2019). van Bommel et al. (2020) reported a chloride increase of 0.2–2.4 mmol/l in 24 h upon treatment with dapagliflozin. Masuda et al. (2020) also reported an increase to 3 mM/Day in urinary chloride compared to 2.3 mM/Day in control after 8 weeks of treatment with ipragliflozin (in rats). For diabetic males and females, we predict a change of 0.11–0.61 mmol/l in plasma chloride (when normalized with urine volume in healthy patients; separate for men and women) or 0.14–0.8 mM/Day. Sha et al. (2014) observed a change of 330 mM/day of urinary glucose excretion upon 30 mg of canagliflozin while their Korean patients showed an increase of 220 mM/day after 24 h. Taking into account the smaller size of Korean patients in comparison to their western counterparts, we note that same dose may show differing effects in patients of different sizes, which we also show with our model (women are generally smaller than men). They also observed a transient change of 5–20% in urine volume after 1 day of dosage ≥200 mg and no change in 24 h urinary excretion. For instance, the model predicts that in the moderate diabetes case, urine output doubles in women, and almost triples in men (Figure 4B). In contrast, empagliflozin increases urine volume by 24% (Mordi et al., 2020). Excessive urine output may result in volume depletion, which in turn activates mechanisms to reduce urine production. The model also predicts higher K^+^ in patients under 500 mg/day remogliflozin (120–300% compared to 60% in trials) and Na^+^ (25–400% compared to 54%) excretion compared to that seen in clinical trials (Kapur et al., 2013; Cherney et al., 2014; Tanaka et al., 2017; Eickhoff et al., 2019). The model assumption for 90% SGLT2 inhibition in the model predicts similar glucose excretion as seen in these trials. Studies suggest that SGLT2 inhibition under hyperglycemic and hyperfiltration conditions increases the load to NKCC2 and SGLT1. This increases Na^+^ at the macula densa and is counterbalanced by tubuloglomerular feedback (Novikov and Vallon, 2016). K^+^ excretion is reported to increase under the influence of diuretics and high urine flow as seen in the model (Udensi and Tchounwou, 2017). In response to hyperkalemia, aldosterone is released to moderate the body’s K^+^ balance (Arroyo et al., 2011). Other trials have shown no change in sodium, potassium and chloride levels and a decrease in urinary volume by 20–40% depending on the dosage (Whaley et al., 2012; Kapur et al., 2013). It should be noted that typically male rodents have significantly larger kidneys and thus, larger transport area than female rodents. Such differences are not seen in humans. Additionally, because most of these mechanisms are external to the human kidney, they are not represented in the present model, which may explain the excessive urine output and excretion predicted by the model.

A major limitation of the present models is that the interstitial fluid composition is assumed to be known *a priori*. As such, the models do not capture the interactions among different nephron segments. A worthwhile extension is to incorporate these interactions and perhaps capture the spatially heterogeneous distribution of the renal tubules (Pannabecker et al., 2008; Layton et al., 2012; Pannabecker and Layton, 2014). Moreover, as previously noted, tubuloglomerular feedback is not explicitly represented. Once data describing that feedback system in humans become available, the present model can be combined with a model of the tubuloglomerular feedback (Chen et al., 2011; Sgouralis et al., 2015), so that changes in SNGFR following drug treatments can be predicted. Besides controlling solute transport, the kidney also filters and secretes uremic toxins such as indoxyl sulfate, hippuric acid, and *p*-cresyl sulfate, important food and drug organic metabolites whose accumulation in the blood induces many complications and ultimately lead to kidney failure (Herget-Rosenthal et al., 2009; Duranton et al., 2012; Schophuizen et al., 2013). Importantly, the uremic toxin transporters such as organic anionic transporter (OAT) 1 and 3 also display sex-differences (Urakami et al., 1999; Groves et al., 2006; Euteneuer et al., 2019). Moreover, evidence shows that the OAT3 expression is reduced in diabetic rats (Lungkaphin et al., 2014; Phatchawan et al., 2014), and as such including uremic toxins and their transporters represents an important avenue for model extension. Despite its limitations, the present models are a stepping-stone to evaluate the renal mechanisms under the effect of commonly prescribed medications besides SGLT2 inhibitors, such as blockers of the angiotensin II system, in both women and men with diabetes, and to assess the impact of impaired kidney function.

## Data Availability Statement

The datasets presented in this study can be found in online repositories. The names of the repository/repositories and accession number(s) can be found below: https://github.com/uwrhu/Python-nephron-model-parallel-code-latest.

## Author Contributions

AL: conceptualization, methodology, software, resources, data curation, supervision, project administration, and funding acquisition. AL and SS: validation, formal analysis, and investigation. SS: visualization. AL, SS, and AC: writing – original draft preparation, writing – review and editing, read, and agreed to the submitted version of the manuscript.

## Conflict of Interest

The authors declare that the research was conducted in the absence of any commercial or financial relationships that could be construed as a potential conflict of interest.

## Publisher’s Note

All claims expressed in this article are solely those of the authors and do not necessarily represent those of their affiliated organizations, or those of the publisher, the editors and the reviewers. Any product that may be evaluated in this article, or claim that may be made by its manufacturer, is not guaranteed or endorsed by the publisher.
