# Supplementary material for: Sex-Specific Computational Models of Kidney Function in Patients With Diabetes
Source: Front Physiol. 2022 Jan 26;13:741121. doi: 10.3389/fphys.2022.741121 (PMC8827383; doi:10.3389/fphys.2022.741121)
Supplement: Supplementary file 2 [file Data_Sheet_1.docx]

**S1** Sensitivity analysis

To assess the sensitivity of model behaviors to changes in key parameters, we conducted a sensitivity analysis, in which we varied the activities of glucose transporters (SGLT2, SGLT1, GLUT2 [12]) and key Na+ transporters NKCC2 [58] and Na+-K+-ATPase [59] that are known to be major determinants of electrolyte excretions and exhibit sex differences. Given the substantial uncertainties in these transporter activities, we varied the corresponding activities individually by +/-20% and determined the effect on urine output, glucose and electrolyte excretion rates. The sensitivity study was conducted in both men and women, under healthy, moderately and severely diabetic conditions, all with SGLT2 inhibition. We have divided the results into two groups: Severe diabetes and moderate diabetes. Figure 8 in the text shows results for the male model with severe diabetes, with a 20% increase in individual transporter activities. The rest of the figures are given below:

1. Severely diabetic men and women:


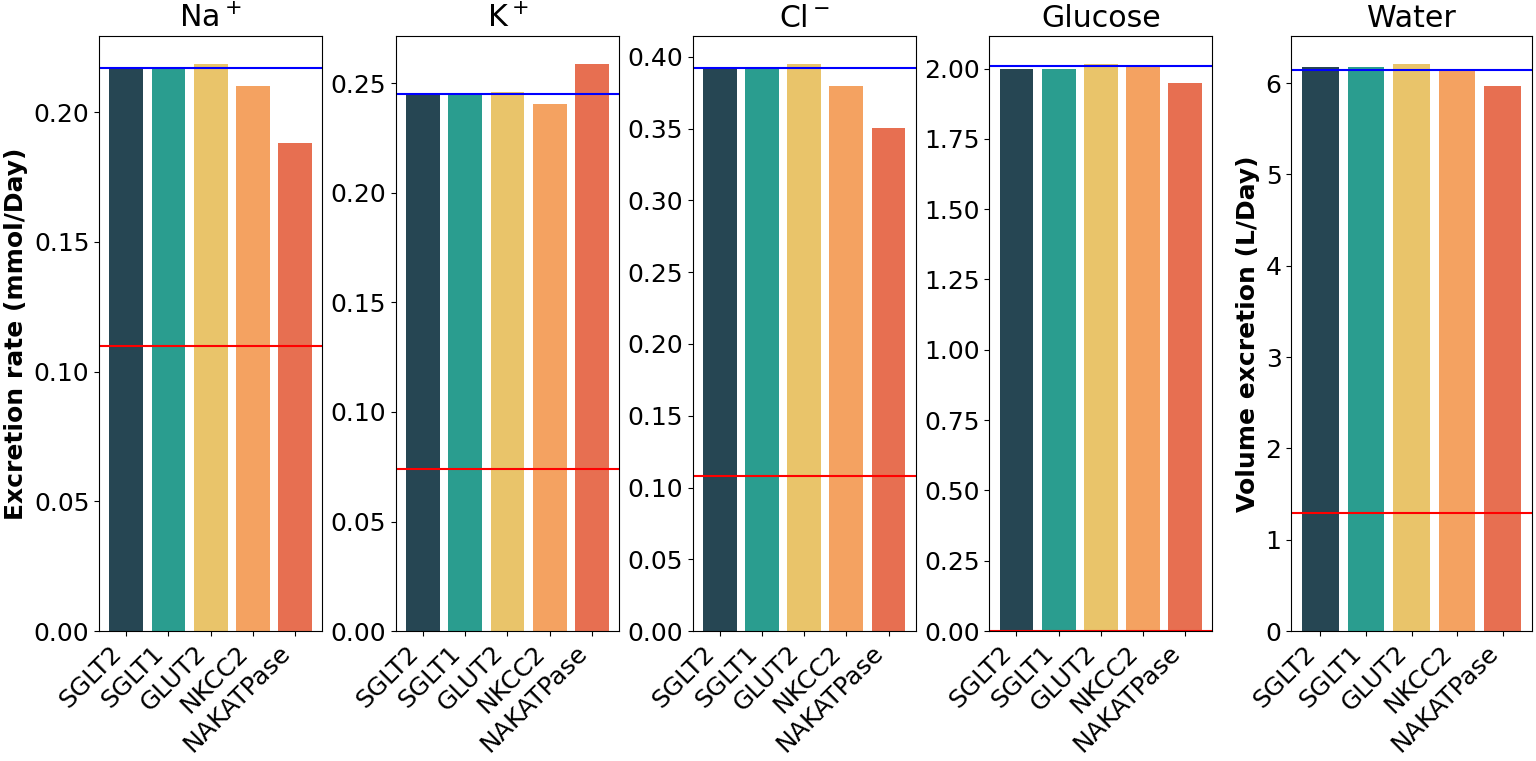


**Figure S1.1**: Urinary excretion of Na^+^, K^+^, Cl^-^ , glucose and fluid, computed for severely diabetic women. Results are obtained with SGLT2 inhibition upon a **20% increase** in the individual activity of the five specific transporters: SGLT2, SGLT1, GLUT2, NKCC2, Na+-K+-ATPase. The red lines denote the solute and volume prediction in healthy female under no drugs. The blue lines indicate the levels predicted by the model for severely diabetic females under 90% SGLT2 inhibition.


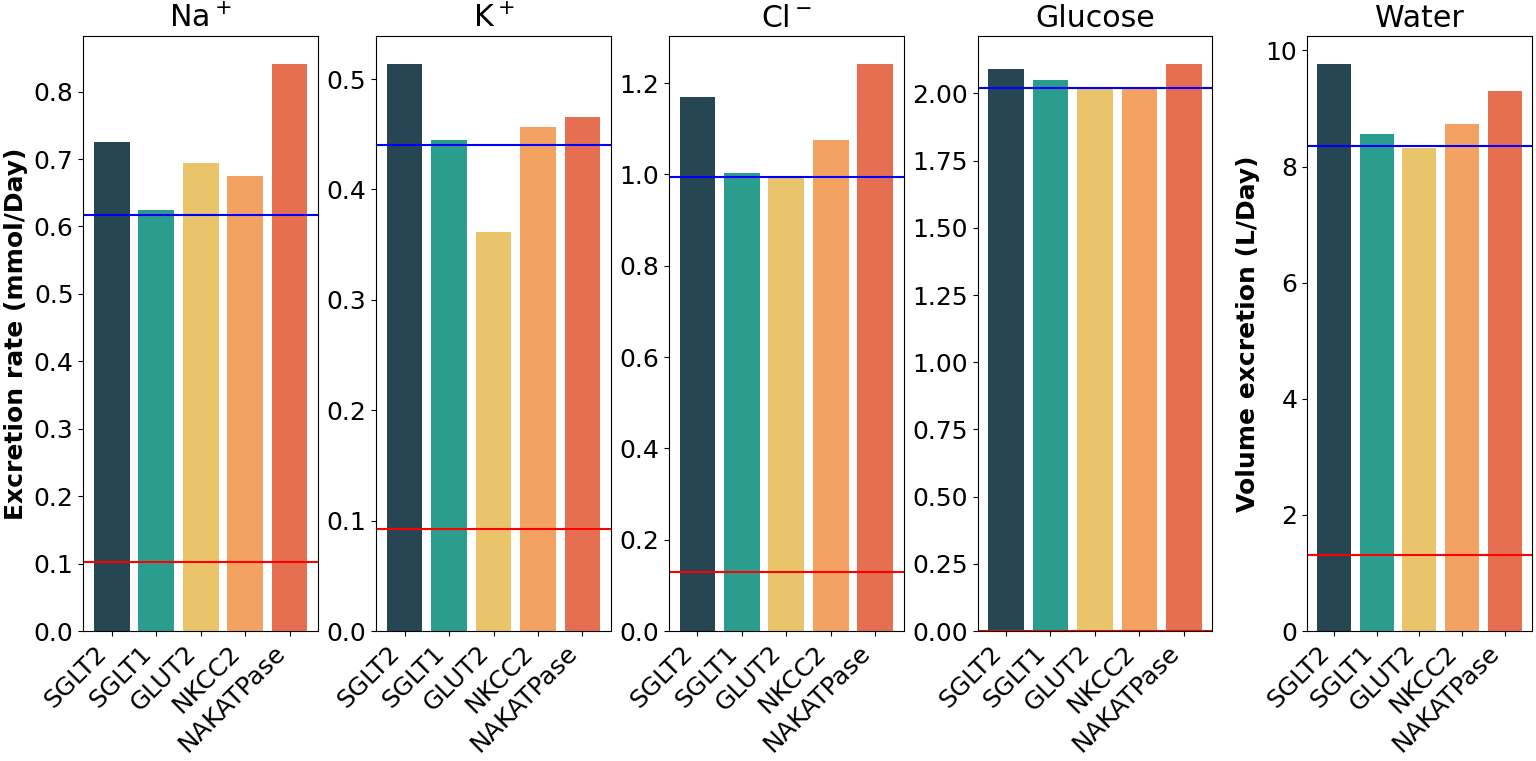


**Figure S1.2**: Urinary excretion of Na^+^, K^+^, Cl^-^ , glucose and fluid, computed for severely diabetic men. Results are obtained with SGLT2 inhibition upon a **20% decrease** in the individual activity of the five specific transporters: SGLT2, SGLT1, GLUT2, NKCC2, Na+-K+-ATPase. The red lines denote the solute and volume prediction in healthy male under no drugs. The blue lines indicate the levels predicted by the model for severely diabetic males under 90% SGLT2 inhibition.


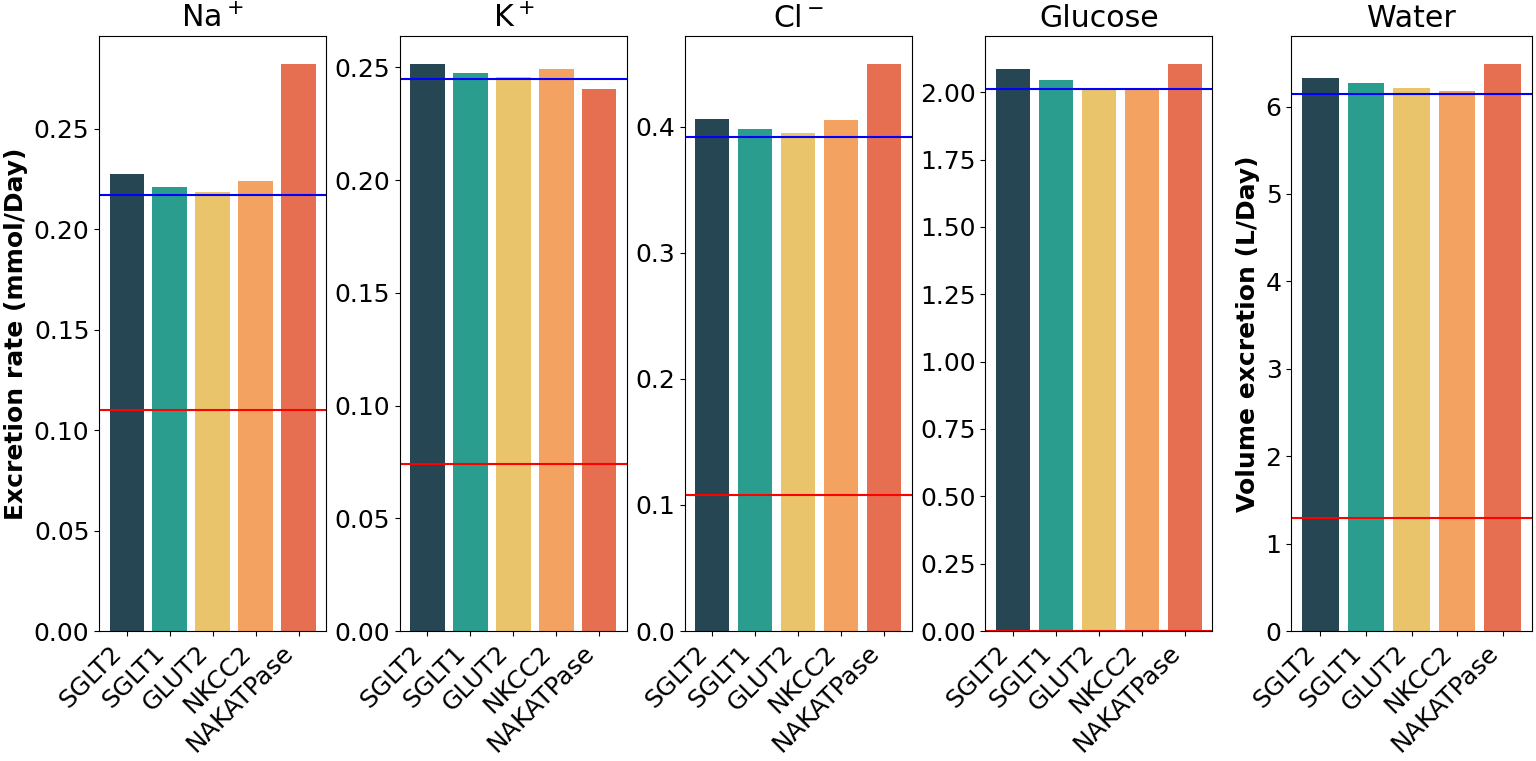


**Figure S1.3**: Urinary excretion of Na^+^, K^+^, Cl^-^ , glucose and fluid, computed for severely diabetic women. Results are obtained with SGLT2 inhibition upon a **20% decrease** in the individual activity of the five specific transporters: SGLT2, SGLT1, GLUT2, NKCC2, Na+-K+-ATPase. The red lines denote the solute and volume prediction in healthy female under no drugs. The blue lines indicate the levels predicted by the model for severely diabetic females under 90% SGLT2 inhibition.

1. Moderately diabetic men and women


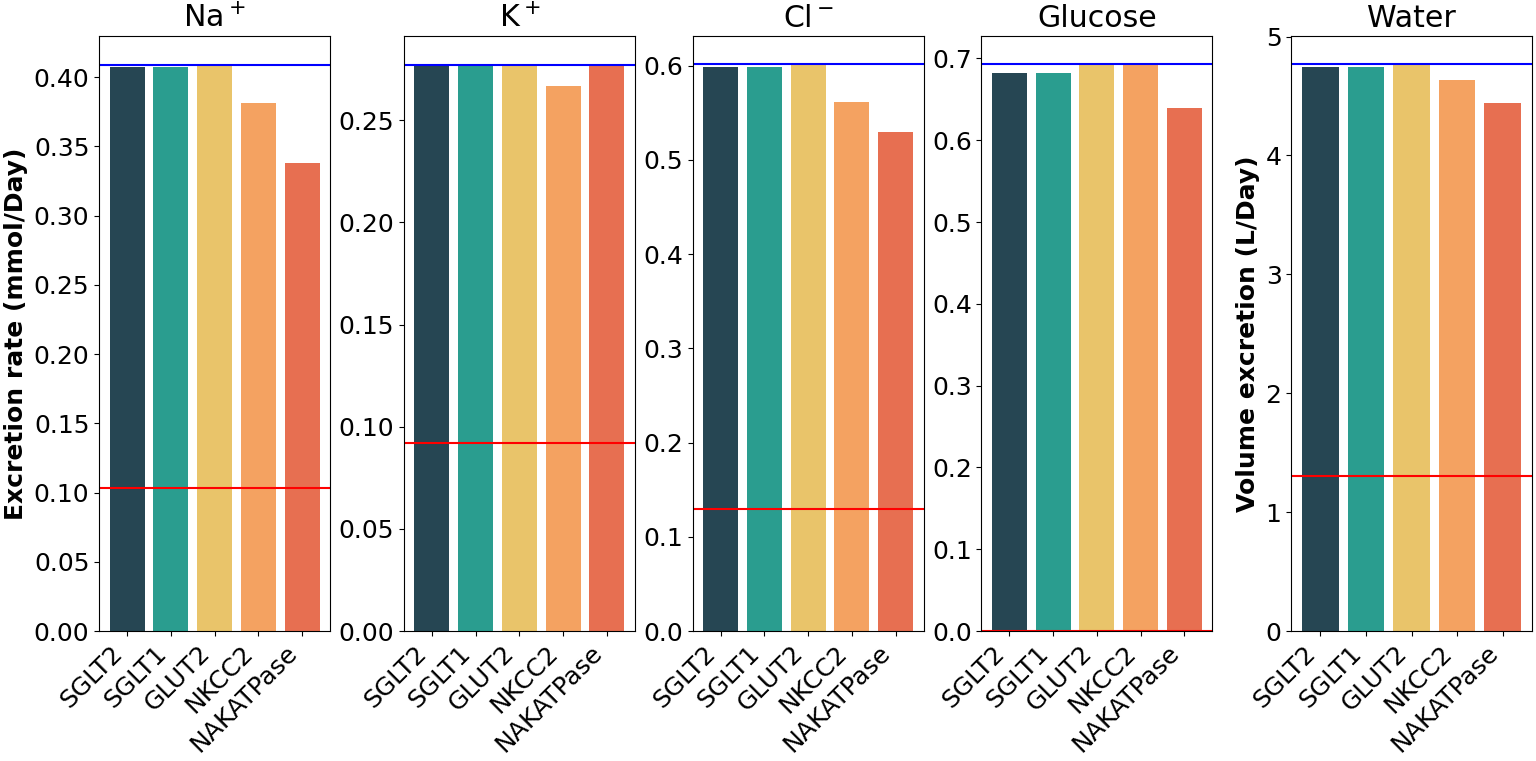


**Figure S1.4**: Urinary excretion of Na^+^, K^+^, Cl^-^ , glucose and fluid, computed for moderately diabetic men. Results are obtained with SGLT2 inhibition upon a **20% increase** in the individual activity of the five specific transporters: SGLT2, SGLT1, GLUT2, NKCC2, Na+-K+-ATPase. The red lines denote the solute and volume prediction in healthy male under no drugs. The blue lines indicate the levels predicted by the model for moderately diabetic males under 90% SGLT2 inhibition.


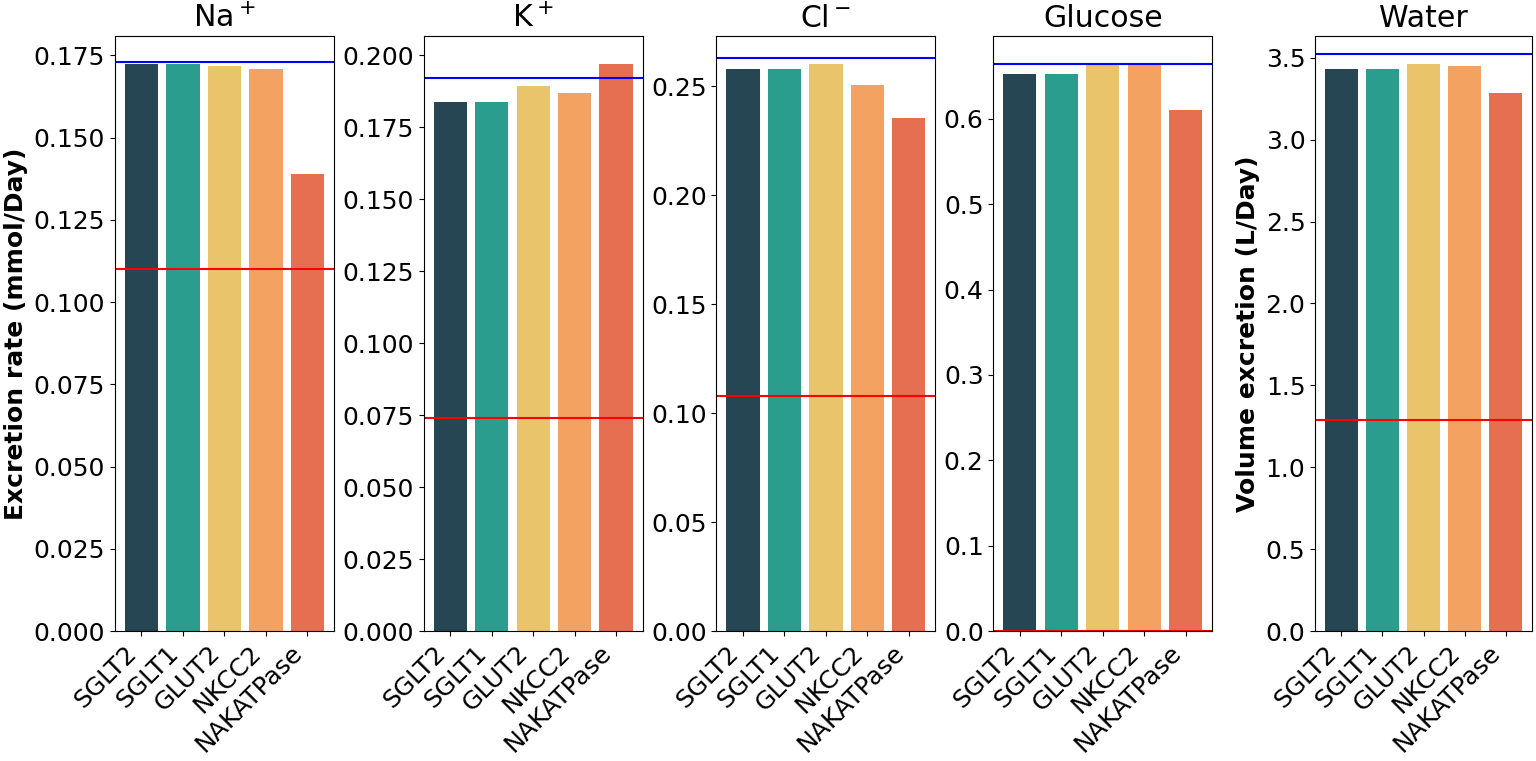


**Figure S1.5**: Urinary excretion of Na^+^, K^+^, Cl^-^ , glucose and fluid, computed for moderately diabetic women. Results are obtained with SGLT2 inhibition upon a **20% increase** in the individual activity of the five specific transporters: SGLT2, SGLT1, GLUT2, NKCC2, Na+-K+-ATPase. The red lines denote the solute and volume prediction in healthy female under no drugs. The blue lines indicate the levels predicted by the model for moderately diabetic females under 90% SGLT2 inhibition.


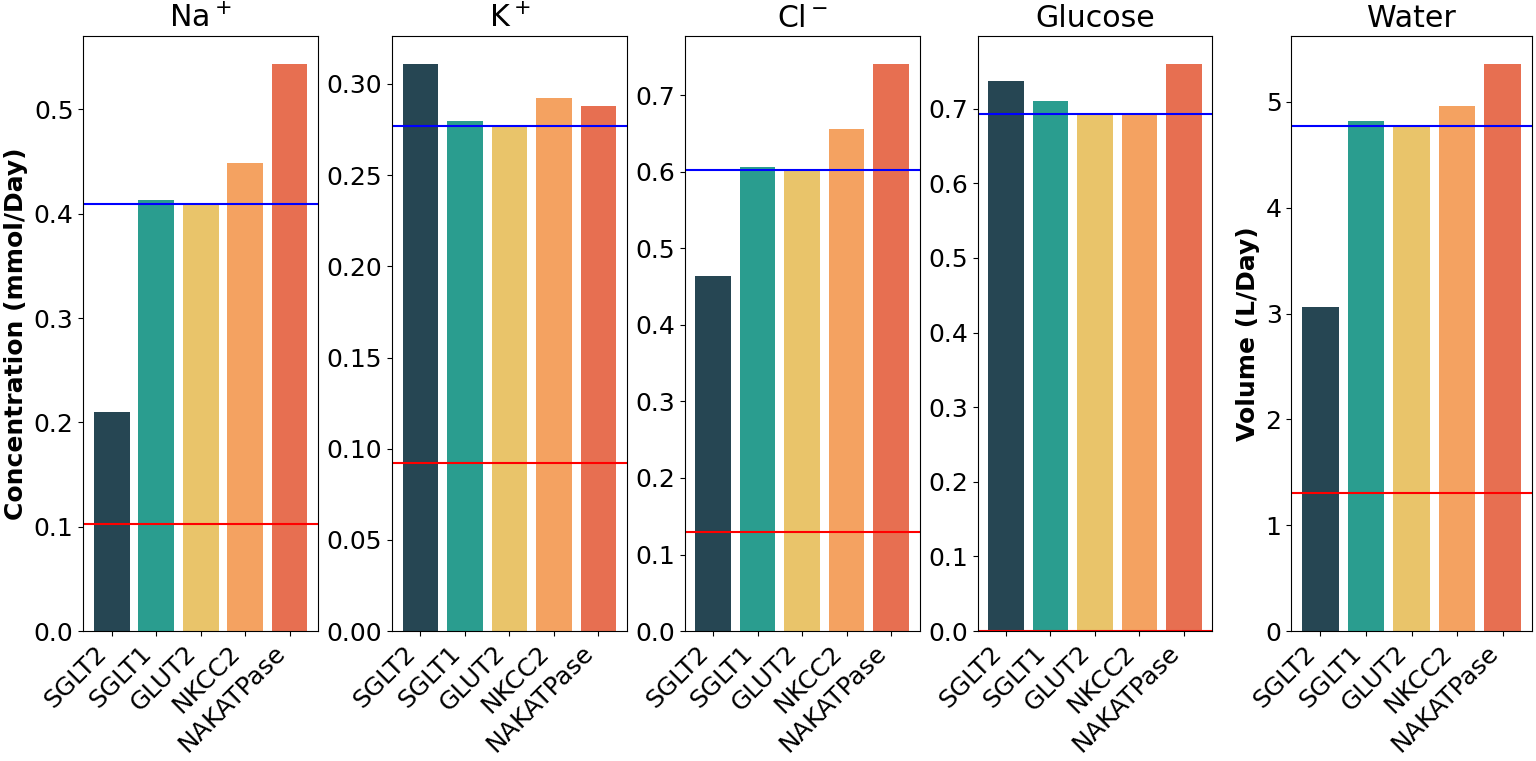


**Figure S1.6**: Urinary excretion of Na^+^, K^+^, Cl^-^ , glucose and fluid, computed for moderately diabetic men. Results are obtained with SGLT2 inhibition upon a **20% decrease** in the individual activity of the five specific transporters: SGLT2, SGLT1, GLUT2, NKCC2, Na+-K+-ATPase. The red lines denote the solute and volume prediction in healthy male under no drugs. The blue lines indicate the levels predicted by the model for moderately diabetic males under 90% SGLT2 inhibition.


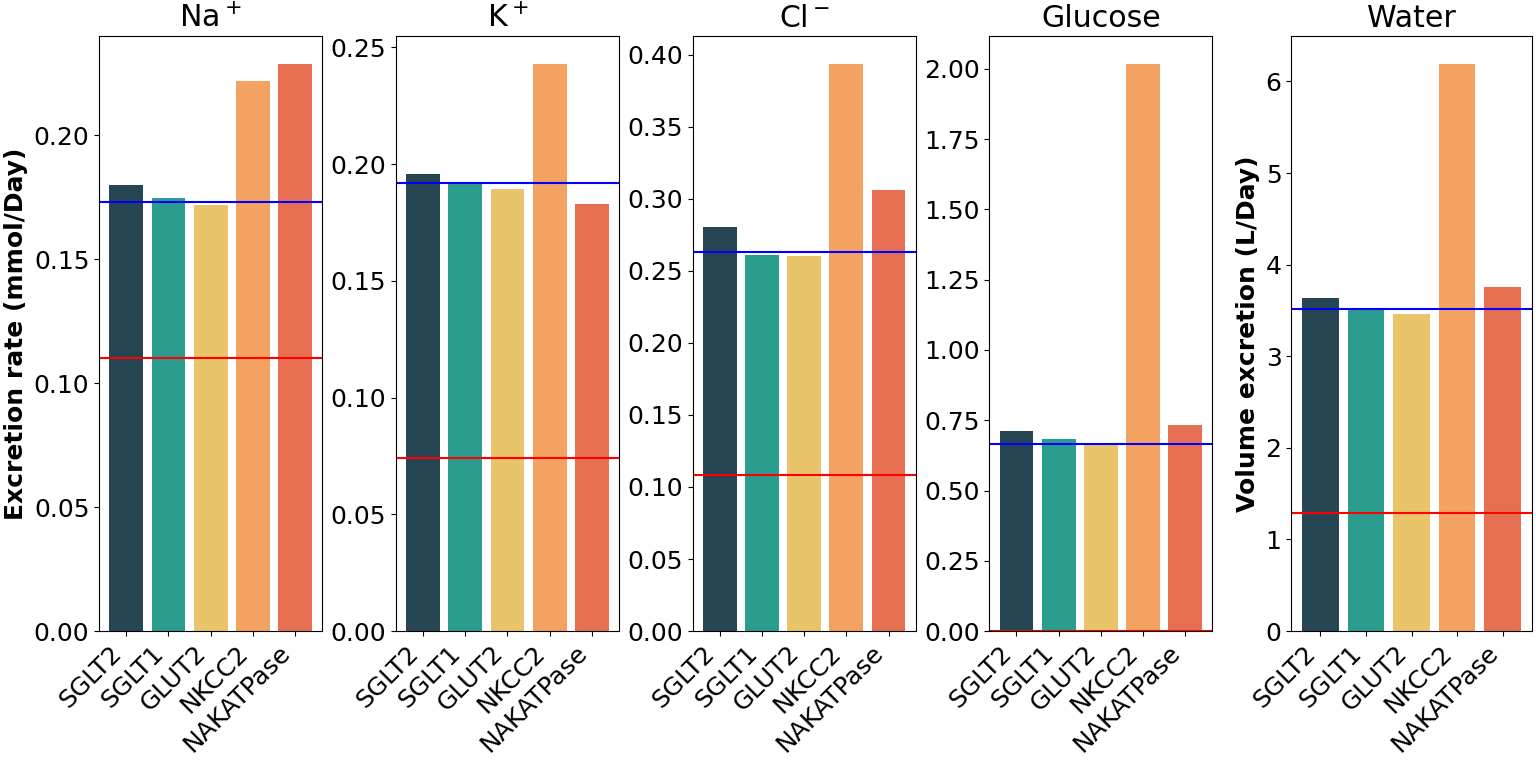


**Figure S1.7**: Urinary excretion of Na^+^, K^+^, Cl^-^ , glucose and fluid, computed for moderately diabetic women. Results are obtained with SGLT2 inhibition upon a **20% decrease** in the individual activity of the five specific transporters: SGLT2, SGLT1, GLUT2, NKCC2, Na+-K+-ATPase. The red lines denote the solute and volume prediction in healthy female under no drugs. The blue lines indicate the levels predicted by the model for moderately diabetic females under 90% SGLT2 inhibition.
